# Baicalin Improves Survival in a Murine Model of Polymicrobial Sepsis via Suppressing Inflammatory Response and Lymphocyte Apoptosis

**DOI:** 10.1371/journal.pone.0035523

**Published:** 2012-05-08

**Authors:** Jiali Zhu, Jiafeng Wang, Ying Sheng, Yun Zou, Lulong Bo, Fei Wang, Jingsheng Lou, Xiaohua Fan, Rui Bao, Youping Wu, Feng Chen, Xiaoming Deng, Jinbao Li

**Affiliations:** Department of Anesthesiology and Critical Care, Changhai Hospital, Second Military Medical University, Shanghai, China; University of Cincinnati, United States of America

## Abstract

**Background:**

An imbalance between overwhelming inflammation and lymphocyte apoptosis is the main cause of high mortality in patients with sepsis. Baicalin, the main active ingredient of the *Scutellaria* root, exerts anti-inflammatory, anti-apoptotic, and even antibacterial properties in inflammatory and infectious diseases. However, the therapeutic effect of baicalin on polymicrobial sepsis remains unknown.

**Methodology/Principal Findings:**

Polymicrobial sepsis was induced by cecal ligation and puncture (CLP) in C57BL/6 mice. Mice were infused with baicalin intraperitoneally at 1 h, 6 h and 12 h after CLP. Survival rates were assessed over the subsequent 8 days. Bacterial burdens in blood and peritoneal cavity were calculated to assess the bacterial clearance. Neutrophil count in peritoneal lavage fluid was also calculated. Injuries to the lung and liver were detected by hematoxylin and eosin staining. Levels of cytokines, including tumor necrosis factor (TNF)-alpha, interleukin (IL)-6, IL-10 and IL-17, in blood and peritoneum were measured by enzyme-linked immunosorbent assay. Adaptive immune function was assessed by apoptosis of lymphocytes in the thymus and counts of different cell types in the spleen. Baicalin significantly enhanced bacterial clearance and improved survival of septic mice. The number of neutrophils in peritoneal lavage fluid was reduced by baicalin. Less neutrophil infiltration of the lung and liver in baicalin-treated mice was associated with attenuated injuries to these organs. Baicalin significantly reduced the levels of proinflammatory cytokines but increased the level of anti-inflammatory cytokine in blood and peritoneum. Apoptosis of CD3^+^ T cell was inhibited in the thymus. The numbers of CD4^+^, CD8^+^ T lymphocytes and dendritic cells (DCs) were higher, while the number of CD4^+^CD25^+^ regulatory T cells was lower in the baicalin group compared with the CLP group.

**Conclusions/Significance:**

Baicalin improves survival of mice with polymicrobial sepsis, and this may be attributed to its antibacterial property as well as its anti-inflammatory and anti-apoptotic effects.

## Introduction

Sepsis and sequential multiple organ failure/dysfunction syndrome (MOF/MODS) remain to be the leading cause of death in intensive care units, despite the progression of medical care [1], [2]. The sepsis response may begin with an infectious nidus. In the early phase of sepsis, overwhelming inflammatory response is initiated after microbial infection [3]. During this stage, cytokine storm is triggered, as well as excessive neutrophils recruitment in multiple organs, complement system activation and coagulation dysfunction. This proinflammatory state has been defined as being a systemic inflammatory response syndrome (SIRS) [4]. The late hypodynamic phase is characterized by T cell hyporesponsiveness, defective antigen presentation and loss of delayed-type hepersensitivity response, which has been recently termed a compensatory anti-inflammatory response syndrome (CARS) [4], [5].

Neutrophil activation during the early phase of sepsis induces organ damage by directly adhering to endothelium which produces microvascular occlusions or releasing proteolytic enzymes and oxygen radicals [6], thus it is reasonable that resolution of inflammation may improve survival of sepsis. However, clinical trials show that a single anti-inflammatory treatment such as neutralization of endotoxin, tumor necrosis factor (TNF) or interleukin (IL)-1, is inefficient or even detrimental to the host, as endogenous cytokines are crucial for bacterial clearance and neutralisation of them may induce immunoparalysis [7]–[9]. Sepsis itself may induce immunosuppression in absence of any anti-inflammatory treatment, as characterized by loss of delayed hypersensitivity, inability to clear the infection and predisposition to secondary infections [10]. Joshi et al. [11] speculated that inflammation and apoptosis are simultaneously activated during sepsis, as phosphorylation of some kinases, such as extracellular regulated protein kinases 1 and p38 mitogen-activated protein kinase 1 (MAPK1), may enhance both proliferation and apoptosis. Therefore, immunomodulation, which reduces inflammatory load while preserving immune function, becomes a promising therapeutic approach for sepsis.

Baicalin (5,6,7-Trihydroxyflavone) is a main active ingredient derived from the dried root of *Scutellaria*, a popular herb in traditional Chinese medicine used to treat fever [12]–[13]. Baicalin, a small-molecule monomer, exhibits anti-inflammatory, anti-oxidant, anti-apoptotic, and antibacterial properties. Studies in rats with severe acute pancreatitis showed that baicalin inhibited inflammation and protected multiple organs function in [14]–[15]. Baicalin was also reported to inhibit lipopolysaccharide (LPS)-induced inflammation both *in vitro* and *in vivo*. *In vitro*, baicalin reduced LPS-stimulated macrophage activation [16], while *in vivo*, it inhibited pro-inflammatory cytokines and nitric oxide (NO) production, nuclear factor-κB (NF-κB) activation, caspase-3 activity, as well as reversed organ injury caused by endotoxic shock [16]–[17]. Moreover, baicalin can even be used as an adjunctive therapy against methicillin-resistant Staphylococcus aureus (MRSA) *ex vivo* and Escherichia coli (E. coli) meningitis in animals [18]–[19]. The aglycone derivative of baicalin, baicalein, is thought to interfere with the cell wall integrity of bacteria by binding to peptidoglycan.

Interestingly, these special pharmacological actions of baicalin are all that are required in treatment of polymicrobial sepsis. Therefore, this study is performed to determine whether baicalin is effective in improving survival of mice with polymicrobial sepsis induced by cecal ligation and puncture (CLP). The potential protective mechanism of baicalin appeares to be associated with its effects on inflammation, apoptosis and microbial clearance.

**Figure 1:**
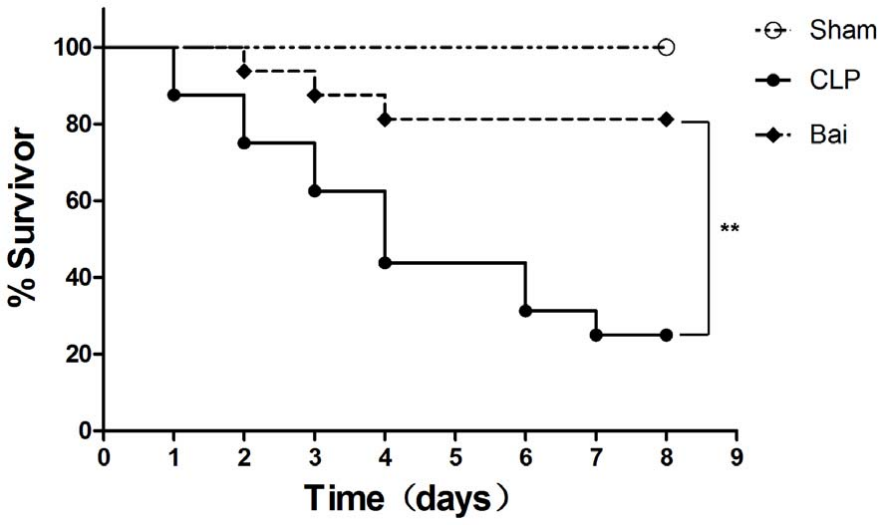
Survival rate of septic mice after injection with baicalin. Baicalin improves survival of polymicrobial sepsis in CLP mice. In the bai group, mice were treated with intra-peritoneal baicalin (100 mg/kg) at 1 h, 6 h and 12 h after CLP. Normal saline of equal volume was administered in sham and CLP groups. Bai, baicalin group; CLP, CLP group. Survival analyses with log-rank test. ** *P*<0.01.

## Methods

### CLP Model of Sepsis

All experiments were approved by the Animal Care and Use Committee of Changhai hospital (permission number:CH 20101215-04). Healthy 8–10-week-old C57BL/6 male mice, weighing 22–30 g, were purchased from the Animals Experimentation Center of Second Military Medical University. All animals were acclimatized under controlled temperature (20±2°C), humidity (60±5%) and 12 h light/12 h dark cycle for 1 week before the experiment.

**Figure 2:**
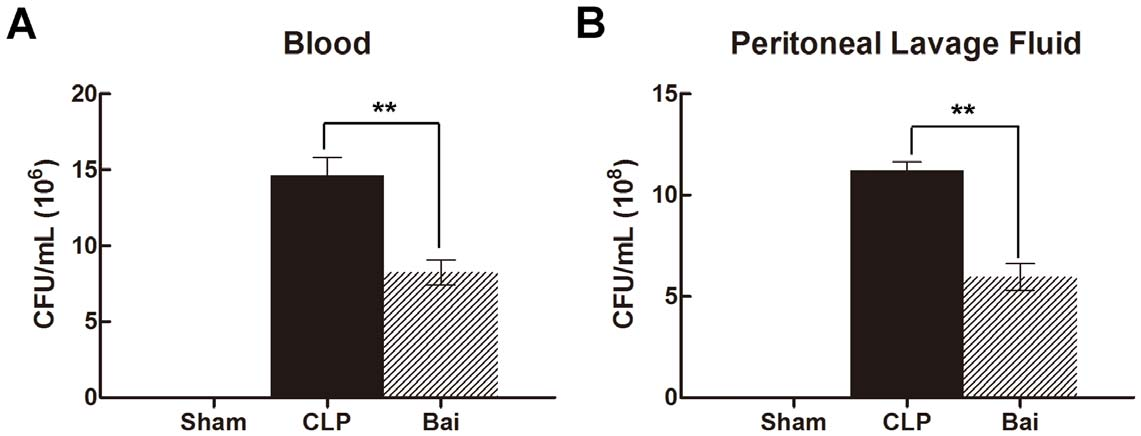
Bacterial clearance in blood (A) and peritoneum (B) in septic mice after baicalin treatment. Baicalin enhanced bacterial clearance in blood (A) and peritoneum (B). Blood and peritoneal lavage fluid were harvested 24 h after CLP. Bai, baicalin group; CLP, CLP group. Data analyses with one-way ANOVA and Newman-Keuls. ** *P*<0.01.

The CLP procedure was performed according to the guidelines of Wichterman et al. [20] Briefly, mice were completely anesthetized with isofluorane and a midline abdominal incision was made after the abdomen was disinfected. The cecum was exposed and ligated below the ileocecal valve with a 1–0 Prolene thread. The ligated cecum was then punctured twice with a 22-gauge needle to induce polymicrobial peritonitis. The cecum was slightly compressed until a small drop of stool appeared. The abdominal wall was closed in 2 layers. For control purposes, sham-operated mice underwent the same procedure, including peritoneum opening and bowel exposing, but without ligation and needle perforation of the cecum. After surgery, the mice were resuscitated by subcutaneous injection of 1 ml sterile physiologic saline solution. All mice were given free access to food and water after recovery from anesthesia.

**Figure 3:**
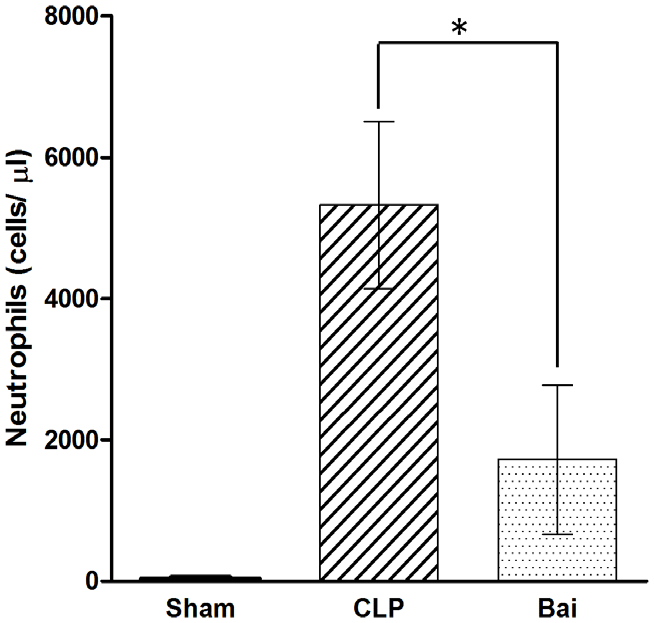
The number of neutrophils in peritoneum in septic mice after administration of baicalin. Baicalin reduced the number of neutrophils in peritoneum. Peritoneal lavage fluid were harvested 24 h after CLP. Bai, baicalin group; CLP, CLP group. Data analyses with one-way ANOVA and Newman-Keuls. * *P*<0.05.

### Drug Administration

Baicalin (Sigma-Aldrich; CAS Number: 21967-41-9; purity, 98%; molecular formula, C21H18O11; molecular weight, 446.36), was dissolved in normal saline and the pH of the solution was adjusted to 7.4 with NaOH. Mice were randomly divided into three groups: (1) sham group: mice underwent the sham operation and received normal saline; (2) CLP group: mice were subjected to CLP and received normal saline; (3) baicalin group: mice were subjected to CLP and treated with baicalin. Baicalin (100 mg/kg, 200 µl) was injected intraperitoneally at 1 h, 6 h, and 12 h following CLP.

**Figure 4:**
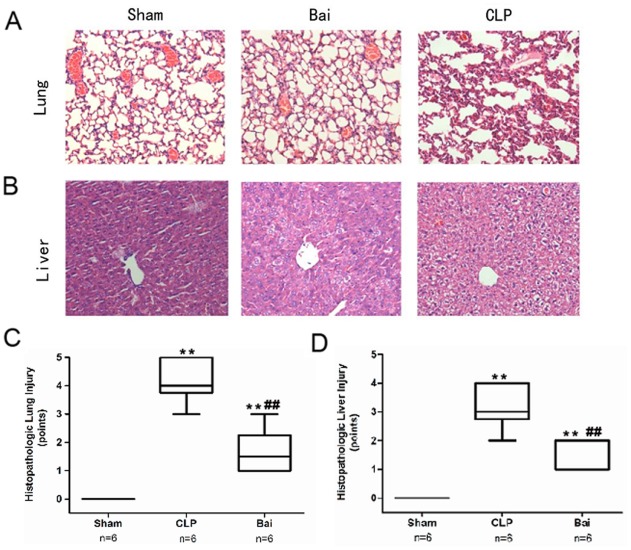
Histopathological changs of septic mice after baicalin treatment. The tissues were harvested 24 h after CLP for histopathologic examination using hematoxylin and eosin staining. Representative images from six animals per group were shown. Histopathological tests showed milder impairment in lung and liver after baicalin administration. A, After baicalin treatment, lung showed less neutrophil accumulation and alveolar destruction. B, Hepatocytes were protected and hepatic sinusoid was also preserved in baicalin treated mice. C, the severity of lung injury was scored as described in Materials and methods. D, the severity of liver injury was scored as above. ** *P*<0.01 vs. sham. ## *P*<0.01 vs. CLP. Bai, baicalin group; CLP, CLP group.

### Effect of Baicalin on the Survival of Septic Mice

In order to compare the effect of baicalin administration at different time points after CLP on survival, mice that had undergone CLP were randomized to receive intraperitoneal baicalin or saline after CLP (n = 16 for each group). Survival rates were assessed over the subsequent 8 days.

**Figure 5:**
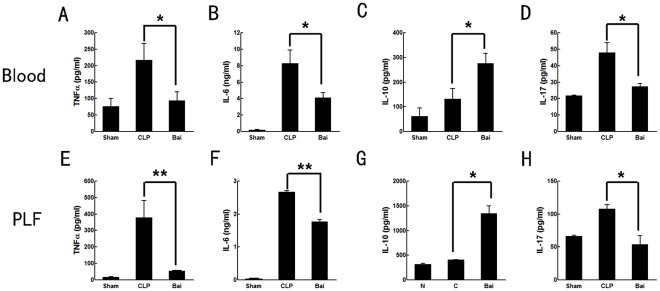
Cytokines expression of septic mice after baicalin injection. Baicalin reduced excretion of proinflammatory cytokines while increased anti-inflammatory cytokine. Samples were collected 24 h after CLP. Proinflammatory cytokines included TNF-alpha, IL-6 and IL-17 (A, B, D in blood and E, F, H in peritoneum). IL-10 represented the anti-inflammatory cytokine (C in blood and G in peritoneum). PLF, peritoneal lavage fluid; Bai, baicalin group; CLP, CLP group. Data analyses with one-way ANOVA and Newman-Keuls. * *P*<0.05, ** *P*<0.01.

### Bacterial Clearance

Blood and peritoneal lavage fluid samples were collected 24 h after surgery. Blood was collected by heart puncture after isoflurane anesthesia (n = 6 for each group). For peritoneal lavage, the skin of the abdomen was cut open in the midline after it was thoroughly disinfected. A total of 2 ml sterile phosphate-buffered saline (PBS) was injected into and aspirated out of the peritoneal cavity several times. Samples of peritoneal lavage fliud were serially diluted to 10, 100, or 1000-fold in 900 µl PBS. Then 100 µl aliquot of each dilution was spread on a tryptic soy agar blood agar plate. All plates were incubated at 37°C for 24 h. Colonies were counted and expressed as colony forming units (CFUs)/ml for all the samples.

**Figure 6:**
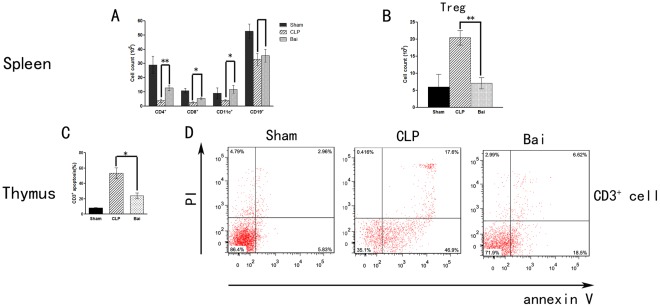
Cell counts in the spleen and apoptosis in the thymus in septic mice after baicalin administration. (A) Baicalin increased the numbers of CD4^+^ and CD8^+^ T lymphocytes, as well as CD11c^+^ dendritic cells, but not CD19^+^ B lymphocytes in spleen. (B) Baicalin reduced the number of regulative T cells in spleen. (C, D) Baicalin inhibited lymphocyte apoptosis in thymus. Bai, baicalin group; CLP, CLP group. Data analyses with one-way ANOVA and Newman-Keuls. *P<0.05, ** *P*<0.01.

### Determination of Neutrophil Count in Peritoneal Lavage Fluid

The peritoneal lavage fluid of the septic and sham-operated mice was harvested 24 h after CLP (n = 6 for each group). After erythrocytes were lysed, cells were stained with fluorochrome-conjugated antibody to cell subset-specific surface marker Gr-1 for neutrophils (Figure S1). Cells were subjected to fluorescence-activated cell sorting (FACS) and cell numbers were calculated by flow cytometry (FCM).

### Histopathological Studies

The lungs and livers of the septic and sham-operated mice were harvested 24 h after surgery for histopathological staining, as previously described. These organ tissues were fixed in buffered formaldehyde (10% in PBS) for more than 8 h, dehydrated in graded ethanol, and embedded in paraffin. Four-micrometer sections were cut and paraffin was removed by xylene. The tissue sections were then stained with the hematoxylin and eosin reagent and observed under light microscopy. The total surface of the slides was scored by two blinded pathologists with expertise in assessing lung and liver tissues. Briey, the criteria for scoring lung inammation were as follows [21]: 0,normal tissue; 1, minimal inammatory change; 2, no obvious damage to the lung architecture; 3, thickening of the alveolar septae; 4, formation of nodules or areas of pneumonitis that distorted the normal architecture; and 5, total obliteration of the field. A semiquantitative method was used to evaluate the degree of liver necrosis: 0, none; 1, individual-cell necrosis; 2, up to 30% lobular necrosis; 3, up to 60% lobular necrosis; 4, more than 60% lobular necrosis. The slides were examined by two pathologists who were unaware of the groups.

### Measurement of Cytokines

Blood and peritoneal lavage fluid samples from the septic or sham-operated mice were collected 24 h after surgery (n = 6 for each group). Plasma and supernatant of peritoneal lavage fluid were collected after centrifugation of the samples (300 g for 5 min). Levels of TNF-alpha, IL-6, IL-10, and IL-17 were measured using the murine enzyme-linked immunosorbent assay (ELISA) kit (Bender Systems, USA) according to manufacturer’s instructions.

### Quantification of Apoptosis in the Thymus

Thymus tissues were harvested from the septic and sham-operated mice 24 h after surgery (n = 6 for each group), and single-cell suspensions were prepared. The annexin V-FITC binding and propidium iodide (PI) staining assay were used to assess apoptosis of CD3^+^ T cells. Stained cells were analyzed via a FACSCalibur and the CellQuest software (Becton Dickinson, USA). T Cells positive for CD3 were first analyzed (Figure S4) and Cells positive for annexin V in CD3^+^ T cells were determined to be apoptotic cells.

### Determination of Cell Counts in the Spleen

The spleens of the septic and sham-operated mice were harvested 24 h after surgery (n = 6 for each group) and single-cell suspensions were prepared. Cells were stained with fluorochrome-conjugated antibodies to cell subset-specific surface markers (CD4, CD8 for T cells, CD4CD25 for Tregs, CD19 for B cells and CD11c for DCs) (Figure S3, Figure S2). The percentage of cells in each group was analyzed by FACS.

### Statistical Analysis

Data are reported as the mean ± SEM. All statistics analyses were performed using Prism 4.0 (GraphPad Software, USA). Survival of the 2 subgroups was estimated by Kaplan-Meier survival curves; comparisons were performed by the log-rank test. All comparisons among groups were performed by Mann-Whitney analysis of variance. For multigroup analysis, intergroup comparisons were performed by Dunn’s test. A significance level of 0.05 was considered to be significant for all calculations.

## Results

### Baicalin Improves Survival in Mice with Polymicrobial Sepsis

In order to investigate whether baicalin was benefit to CLP mice, survival was first assessed at day 8 after CLP. As a result, the survival rate in the CLP group (25%) was lower than that in the sham group (100%; *P*<0.05). Then after baicalin solution treatment, mice in the baicalin group showed an improved 8-day survival (81.25%) compared with the CLP group (25%; *P*<0.05). (Figure 1).

### Baicalin Enhances Bacterial Clearance in Blood and Peritoneum

Blood and peritoneal lavage fluid samples were collected 24 h after CLP. Increased bacterial burden was found in the blood and peritoneal lavage fluid samples in the CLP mice compared with the sham mice. These data demonstrated that mice treated with baicalin after CLP showed a significantly reduced bacterial burden in both blood and peritoneal lavage fluid compared with the CLP mice (*P*<0.05) (Figure 2)**.**

### Baicalin Reduces the Number of Neutrophils in Peritoneum

As neutrophils act as the first line of defense against microbial invasion and the counts of neutrophils migrated to abdominal reflected their migration ability, we assessed the number of neutrophils in the abdominal area. Compared with the CLP group, baicalin significantly decreased the number of neutrophils in peritoneal cavity (*P*<0.05) (Figure 3).

### Histopathological Tests Show Milder Impairment in the Lung and Liver after Baicalin Administration

Lung and liver injury was demonstrated by H&E staining. In CLP mice, lungs were characterized by a large accumulation of neutrophils, with lots of exudates of red blood cells as well as impaired alveoli. The livers of CLP mice were characterized by swollen hepatocytes and no hepatic sinusoids. Administration of baicalin resulted in significant attenuation of these findings, with characteristics similar to those of sham-operated mice, and also reduced the lung and liver injury scores (Figure 4).

### Baicalin Reduces Release of Proinflammatory Cytokines While Increasing Release of Anti-Inflammatory Cytokine

Plasma and peritoneal cytokines levels were assessed to determine the inflammation status of the mice. In CLP mice, levels of pro-inflammatory factors IL-6 and TNF-alpha were significantly increased, while the anti-inflammatory factor IL-10 was decreased in sepsis in both the plasma and peritoneal cavity 24 h after surgery. After baicalin was treated, levels of IL-6 and TNF-alpha were markedly reduced while the level of IL-10 was elevated. IL-17A is an important chemotactic cytokine for neutrophils and plays a detrimental role in sepsis. As expected, baicalin treatment reduced the level of IL-17A in plasma and peritoneal lavage fluid compared with the CLP group (*P*<0.05) (Figure 5).

### Cell Counts in the Spleen and Apoptosis in the Thymus

CLP-induced immunosuppression is characterized by increased apoptosis and hence reduced lymphocyte counts. Baicalin significantly increased the counts of CD4^+^ T lymphocytes, CD8^+^ T lymphocytes and DC cells in the spleen compared with the CLP group (*P*<0.05), with no remarked difference in B cells (CD19^+^) counts between the 2 groups (*P>*0.05). Treg (CD4^+^CD25^+^) counts were lower in the baicalin group compared with the CLP group (*P*<0.05) (Figure 6). In the thymus, mice treated with baicalin had a significant reduction in the number of apoptotic CD3^+^ T lymphocytes compared with the CLP group (*P*<0.05) (Figure 6).

## Discussion

This study for the first time examined the effects of baicalin on polymicrobial sepsis induced by CLP. Baicalin administration had been suggested to protect lung and liver, enhance bacterial clearance, and improve survival in sepsis in our study. It was interesting to notice that the therapeutic effect of baicalin in sepsis seemed to be associated with several different aspects of sepsis, including attenuated excessive inflammatory response, preserved adaptive immune reaction and enhanced bacterial clearance. According to our present study, baicalin inhibited neutrophil mobilization and promoted a shift from pro-inflammatory to anti-inflammatory response. On the other hand, apoptosis of T cells in thymus was markedly inhibited following baicalin treatment. In line with these findings, baicalin enhanced the numbers of splenic T cells and DC cells and inhibited the production of Tregs (CD4^+^CD25^+^ T cell).

It is generally accepted that uncontrolled inflammation is the main factor contributing to organ dysfunction and death during sepsis [6], [22]. After entry into the host, the pathogens is recognized by pathogen recognition receptors (PRRs, which are receptors recognizing pathogen- or danger-associated molecular patterns, such as Toll-like receptors), leading to activation of intracellular signaling, such as NF-κB, and further amplifying the inflammatory responses [23]. Neutrophils, abnormally activated by the overwhelming cytokine production, release proteolytic enzymes and reactive oxygen species (ROS), which finally lead to multiple organ dysfunction [6]. Circulating pro-inflammatory mediators are always greatly upregulated in septic patients and some of them are considered to be diagnostic and prognostic biomarkers, such as C-reactive protein (CRP), IL-6 and IL-8 [24]–[25]. Studies on genetic polymorphism also showed higher severity and mortality in septic patients that harbor a genotype expressing higher levels of TNF-alpha, interferon-gamma, IL-1, and IL-6 [26]–[27]. Here we demonstrated that baicalin, as an anti-inflammatory agent, reduced neutrophil migration as well as circulating and peritoneal pro-inflammatory factors. For example, we noted that baicalin reduced the level of IL-17A in blood and peritoneum. IL-17A is a proflammatory cytokine and is a mediator of neutrophil stimulation and mobilization by T lymphocytes in sepsis [28]. Yang Ji et al. reported that baicalin suppressed RORγt-mediated IL-17 expression in established T_H_.17 cells [29], which is consistent with our study. Baicalin also greatly improved organ integrity. Zhang et al. reported similar results in a rat model of severe acute pancreatitis [15]. They found that after treating rats with baicalin, levels of IL-1β, thromboxane B2, and phospholipase A2 in blood were significantly reduced, and inflammation of pancreas was greatly attenuated. Thus, baicalin can protect organ function against overwhelming inflammatory response during sepsis.

Althought anti-inflammatory treatment of sepsis is associated with increased risk of inability to eliminate the offending bacteria, we found that baicalin treatment enhanced bacterial clearance. We found a smaller number of peritoneal neutrophils in baicalin-treated mice, and although this result might reflect a compromised innate immune function, it was recently identified that neutrophils were dispensable for defense against bacterial infection [30]. That study showed that fewer neutrophils migration was compensated by macrophages or adaptive immune responses. Enhanced adaptive immune function might be a critical factor contributing to the therapeutic effect of baicalin against sepsis, as demonstrated by an increased number of T lymphocytes as well as reduced lymphocyte apoptosis and Treg levels in the spleen. Massive apoptosis of lymphocytes is one of the main characters of patients with severe sepsis and renders the patients susceptible to secondary infection. Inhibition of lymphocyte apoptosis has been proposed as a promising treatment for sepsis [31] and multiple studies have demonstrated that anti-apoptotic agents improved survival in septic mice [32]–[35]. Therefore, inhibition of lymphocyte apoptosis by baicalin is crucial for its therapeutic effect despite of the attenuated inflammatory responses.

However, the mechanism underlying the anti-apoptotic effect of baicalin remains unclear. Compromised inflammatory response might be one of the causes. At a very early stage of sepsis, although baicalin blocked initiation of inflammatory responses, neutrophil- or macrophage-derived cytokines, such as TNF-alpha, ROS, and NO, might trigger apoptosis [36]. Moreover, baicalin was shown to be capable of prevent apoptosis in LPS-induced cardiomyocyte apoptosis and ischemia-induced neuron apoptosis by inhibiting caspase-3 activity [17]. Baicalin might inhibit lymphocyte apoptosis by attenuating uncontrolled inflammatory responses or directly blocking caspase-3.

It is reported that polymicrobial sepsis increases the number of splenic CD4^+^CD25^+^ Tregs after CLP [37]. Tregs suppress effector cells activity, weakening the body’s immune founction during sepsis. However, baicalin exhibits an important protective effect in sepsis induced by CLP. Baicalin treatment weakens the negative regulatory role of Tregs by reducing the number of Tregs or another mechanism altogether. Specific pathways will be further explored in future.

It was amazing that baicalin acted as a synergistic agent with antibiotics against bacteria in several studies *in vitro*, including *vancomycin-resistant Enterococcus* and MRSA [18], [38]. Baicalin might bind to peptidoglycan on the surface of bacteria and interfere with the integrity of cell wall, much like a cell wall-active antibiotic. Another study also demonstrated that when baicalin was given as an adjunctive therapy to ampicillin, it defended the host against E. coli-induced meningitis *in vivo* [19]. According to the present study, baicalin itself might have an anti-bacterial activity in the treatment of sepsis.

The major limitation of this study is that we have not progressively explored the specific mechanism underlying the anti-inflammatory and anti-apoptotic effects of baicalin. Since there is hardly any efficient treatment against sepsis up to now, baicalin may be a promising agent in the treatment of this potentially fatal condition. Further studies should aim to uncover the mechanism underlying the anti-inflammatory and anti-apoptotic effects of baicalin, as well as to examine the efficacy of baicalin in patients in the clinical setting.

In conclusion, based on the results of this study, baicalin seems to be effective in treating sepsis in mice. Baicalin improves survival of mice with polymicrobial sepsis, which may be attributed to its antibacterial property as well as its anti-inflammatory and anti-apoptotic effects.

## Supporting Information

Figure S1 **Gating on Gr-1^+^ neutrophils in peritoneum.** Compared with the CLP group, the number of Gr-1^+^ neutrophils in mice treated with baicalin was decreased.
(TIF)

Figure S2 **Gating on CD11c^+^ DCs and CD19^+^ B cells in the spleen.** Baicalin increased the numbers of CD 11c^+^ dendritic cells, but not CD19^+^ B lymphocytes in the spleen.
(TIF)

Figure S3 **Gating on CD4^+^ T, CD8^+^ T and CD4^+^CD25^+^ T Cells (Tregs) in the spleen.** Baicalin increased the numbers of CD4^+^ and CD8^+^ T lymphocytes, but reduced the number of regulative T cells in the spleen.
(TIF)

Figure S4 **Gating on CD3^+^ T cells in the thymus.** Apoptosis of CD3^+^ T cells in the thymus was inhibited after treatment with baicalin.
(TIF)
