# Supplementary figures and images for: Baicalin Improves Survival in a Murine Model of Polymicrobial Sepsis via Suppressing Inflammatory Response and Lymphocyte Apoptosis
Source: PLoS One. 2012 May 8;7(5):e35523. doi: 10.1371/journal.pone.0035523 (PMC3348138; doi:10.1371/journal.pone.0035523)

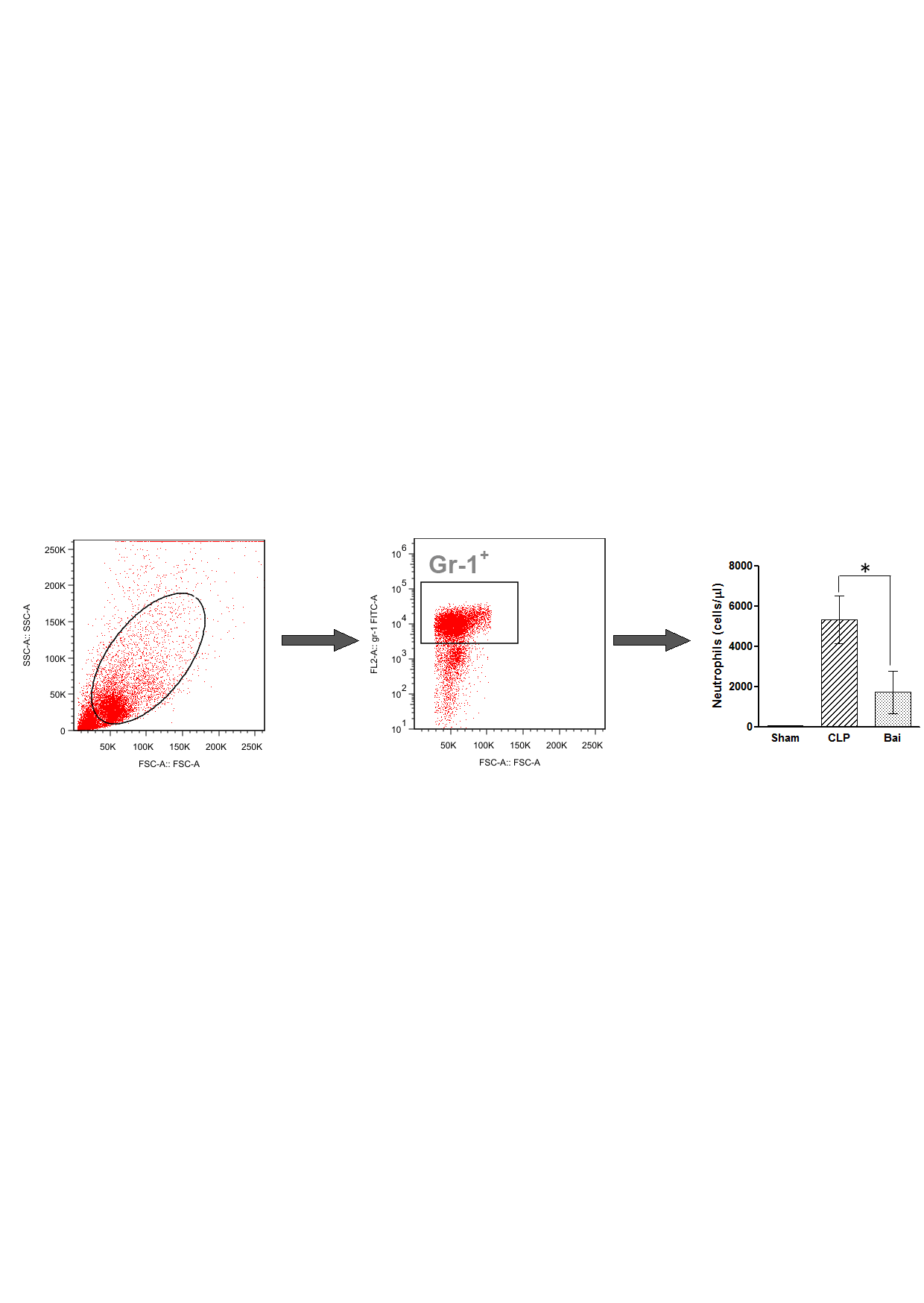

Supplement: Figure S1 — Gating on Gr-1+ neutrophils in peritoneum. Compared with the CLP group, the number of Gr-1+ neutrophils in mice treated with baicalin was decreased. (TIF) [file pone.0035523.s001.tif]

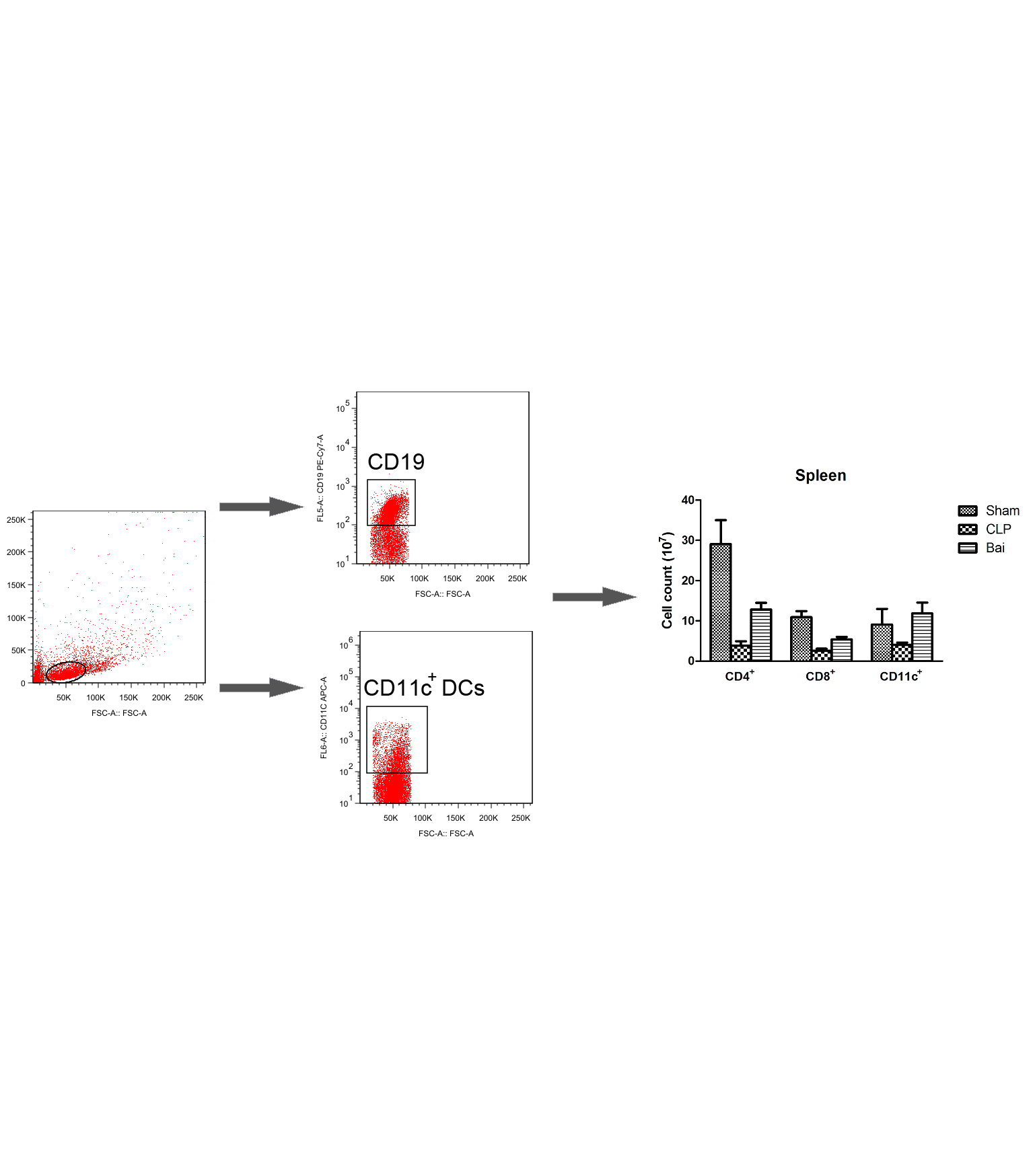

Supplement: Figure S2 — Gating on CD11c+ DCs and CD19+ B cells in the spleen. Baicalin increased the numbers of CD 11c+ dendritic cells, but not CD19+ B lymphocytes in the spleen. (TIF) [file pone.0035523.s002.tif]

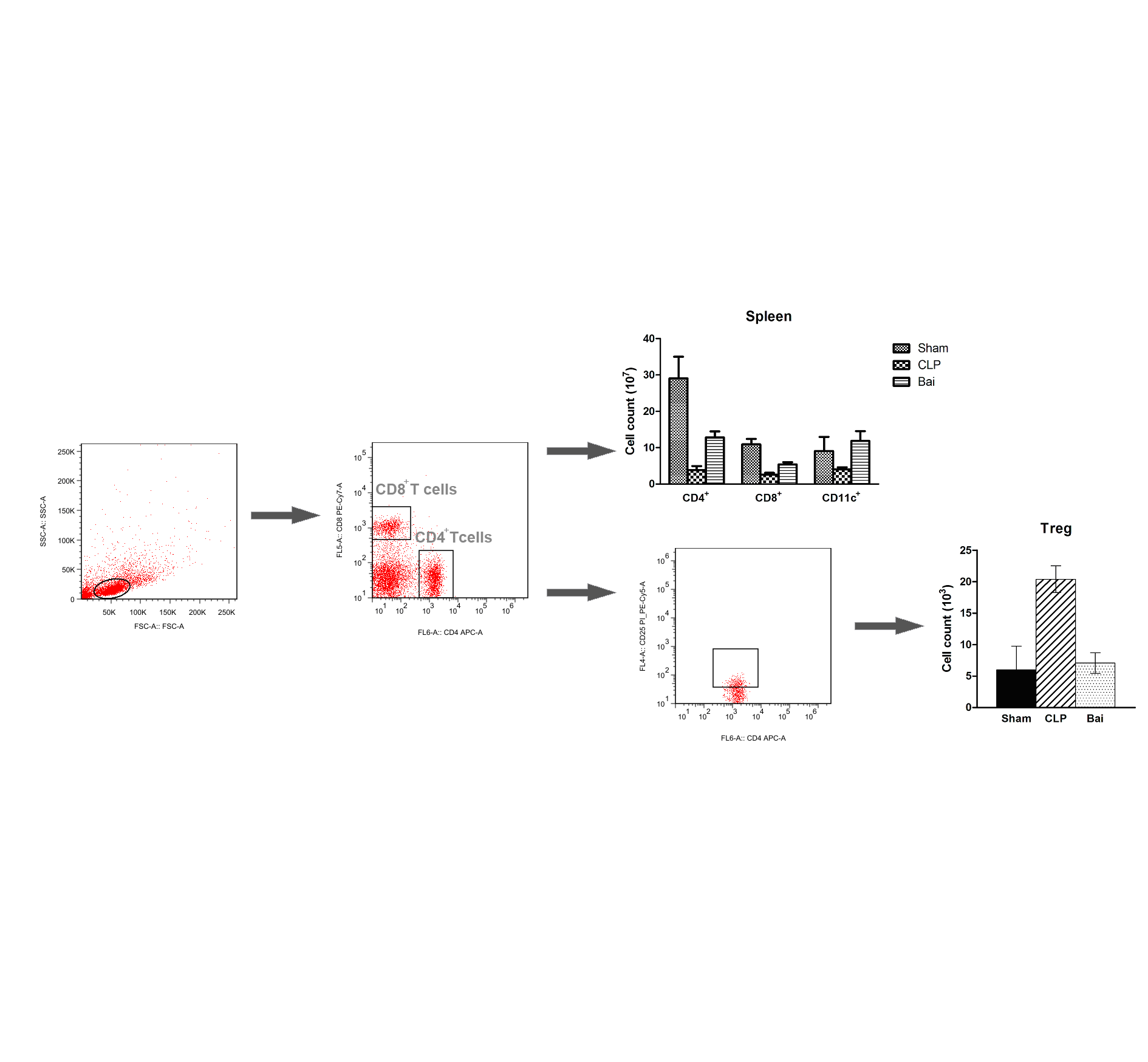

Supplement: Figure S3 — Gating on CD4+ T, CD8+ T and CD4+CD25+ T Cells (Tregs) in the spleen. Baicalin increased the numbers of CD4+ and CD8+ T lymphocytes, but reduced the number of regulative T cells in the spleen. (TIF) [file pone.0035523.s003.tif]

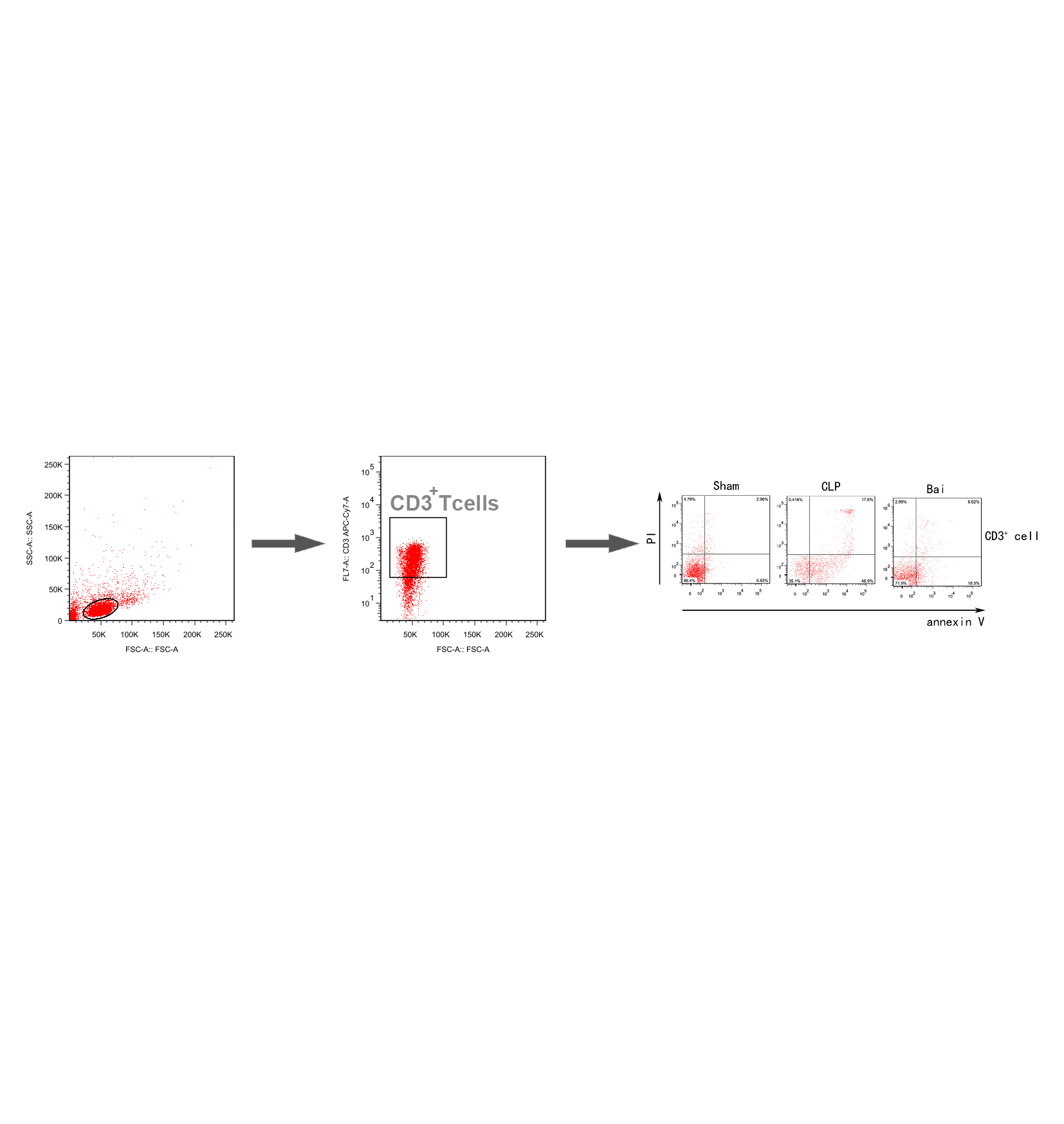

Supplement: Figure S4 — Gating on CD3+ T cells in the thymus. Apoptosis of CD3+ T cells in the thymus was inhibited after treatment with baicalin. (TIF) [file pone.0035523.s004.tif]
